# Challenges in the Treatment of Adolescent Anorexia Nervosa – Is Enhanced Cognitive Behavior Therapy The Answer?

**DOI:** 10.3389/fpsyt.2015.00148

**Published:** 2015-10-14

**Authors:** Tanja M. Legenbauer, Adrian Meule

**Affiliations:** ^1^Hospital for Child and Adolescent Psychiatry, LWL University Hospital of the Ruhr-University Bochum, Hamm, Germany; ^2^Department of Psychology, Center for Cognitive Neuroscience, University of Salzburg, Salzburg, Austria

**Keywords:** enhanced cognitive behavior therapy, anorexia nervosa, eating disorders, treatment

## Relevance and Definition of Anorexia Nervosa

Anorexia nervosa (AN) is among the most common clinical problems encountered by adolescent girls and young women (1). Incidence rates are highest for females aged 15–19 years as they constitute about 40% of all cases (2). Individuals with AN restrict their food and energy intake significantly, which results in a marked low body weight. Despite the low weight, they see and feel their own body as being fatter than it is and have an intense fear of becoming fat (3). Besides the restriction of food intake, these two core symptoms of body image disturbances are included in the diagnostic criteria in the fifth version of the Diagnostic and Statistical Manual of Mental Disorders (4).

## Challenges in the Treatment of Adolescent AN

As therapy outcome seems much more favorable in youth than in adults (5), it is important to optimize treatment strategies within this age group. A variety of treatment options emerged within the last decades, including various specialized in- and outpatient treatments (6). However, despite these efforts and better treatment outcome in adolescents than in adults, long-term treatment success is still unsatisfactory. Although about 50–73% of patients remit, approximately 20–30% remain chronically ill and 9–14% die (5, 7). Inpatient care is required for those in need of intensive treatment, for example, when previous (outpatient) treatment has failed, when there is a rapid decline in weight, or when there are physical conditions that need medical attention (e.g., hemodynamic instability or electrolyte abnormalities) (6). Of those admitted to a hospital, almost 50% of adolescent inpatients need more than one inpatient treatment, 40% of which need three or more (8). Some studies report relapse rates of 85% and more in adolescent patients when reassessed 2–7 years after inpatient treatment (9). In sum, considering the negative impact of hospitalization on the development in adolescence and the risk of chronicity and morbidity, new and effective treatments are urgently needed.

In contrast to AN treatment research in adults, empirical evidence for adolescent treatment is still scarce and mostly of observational nature. Yet, empirical evidence for adolescent treatment has evolved during the last decade. For example, there are some studies, in which family based treatments were compared with treatments focusing on the individual patient. However, as only few studies explored this topic, findings about a superiority of family based interventions over individualized treatments are inconclusive (10–12). Yet, family based treatments may confer better protection against relapse in the long-term (10) and various current practice guidelines recommend them for adolescent AN patients [e.g., Ref. (13)].

In recent years, several innovative treatment approaches have been developed with the aim of solving the problem of low treatment response. Primarily, these include interventions, which have been evaluated in adults and, then, have been adapted to the specific needs of adolescent AN patients. For instance, cognitive-behavioral therapy (CBT), which is a frequently applied transdiagnostic treatment form of eating disorders, including AN (14, 15), emerges as a promising treatment in adolescent AN patients as well. Based on the experiences with non-responding patients, Fairburn broadened the treatment rational of his transdiagnostic approach and established the so-called enhanced cognitive-behavioral therapy (CBT-E) for eating disorders (14, 15).

## Enhanced Cognitive Behavior Therapy for Adolescent Patients with Anorexia Nervosa

Research about CBT-E in eating disorders is rapidly accumulating in recent years (16–27); yet, most studies involved CBT-E as outpatient treatment in adults (Table 1). In a recent article (28), Dalle Grave and colleagues examined the short- and long-term effects of 20-week adapted CBT-E treatment in adolescent AN inpatients, which is the first study that evaluated a form of CBT-E in this population and treatment setting (Table 1). Although sample size was small (*n* = 27) and most patients received post-discharge outpatient treatment, this study revealed promising findings about treatment acceptance and long-term efficacy. Ninety-six percent of patients completed inpatient treatment despite its goal of complete weight restoration. Importantly, body mass markedly increased and eating disorder symptomatology markedly decreased in the course of treatment and these changes were maintained at 1-year follow-up. For example, 83% of patients still had normal weight 1 year after treatment (28).

**Table 1:** **Overview of studies involving enhanced cognitive behavior therapy (CBT-E) in eating disorder patients**.

Study	Treatment setting	Eating disorder diagnoses	Age group	Main results
Allen et al. (16)	Outpatient	AN, BN, EDNOS	Adult	CBT-E and CBT-E preceded by four sessions of motivation-focused therapy did not differ in treatment completion rates and treatment completers in both conditions reported comparable reductions in eating disorder symptoms over time
Byrne et al. (17)	Outpatient	AN, BN, EDNOS	Adult	Fifty-three percent of patients completed treatment, two-thirds of which achieved full or partial remission; longer waiting time for treatment was associated with dropout
Carter et al. (18)	Outpatient	AN, BN, EDNOS	Adult	Fifty-five percent of patients completed treatment; lowest reported weight, the tendency to avoid affect, and time spent on the wait list for treatment predicted dropout
Dalle Grave et al. (19)	Outpatient	AN	Adolescent	Two-thirds of patients completed treatment; weight increased substantially while eating disorder psychopathology decreased and there was little change at 60-week follow-up
Dalle Grave et al. (28)	Inpatient	AN	Adolescent	Ninety-six percent of patients completed treatment; weight, eating disorder symptomatology, and general psychopathology improved substantially and these changes were well maintained at 12-month follow-up
Fairburn et al. (20)	Outpatient	BN, BED, EDNOS	Adult	Patients treated with the focused or broad version of CBT-E exhibited substantial and equivalent change in symptom severity, which was well maintained at 60-week follow-up; treatment outcome did not depend on eating disorder diagnosis; patients with marked mood intolerance, clinical perfectionism, low self-esteem, or interpersonal difficulties appeared to respond better to the more complex treatment, with the reverse pattern evident among the remaining patients
Fairburn et al. (21)	Outpatient	AN	Adult	Sixty-four percent of patients completed treatment; weight and eating disorder symptomatology improved substantially and there was little deterioration at 60-week follow-up
Garte et al. (22)	Day treatment	AN, BN, EDNOS	Adult	Seventy-six percent of patients completed treatment, 32% of which achieved recovery as defined by normal weight status and low scores on the EDE-Q; patients with BN showed greater improvements in eating disorder symptomatology, state anxiety, and self-esteem as compared to patients with AN or EDNOS
Poulsen et al. (23)	Outpatient	BN	Adult	After 5 months and 2 years, more patients receiving CBT-E (42 and 44%) had stopped binge eating and purging as compared to patients receiving psychoanalytic psychotherapy (6 and 15%); by the end of both treatments, substantial improvements in eating disorder features and general psychopathology were observed, but in general these changes took place more rapidly in the CBT-E group
Raykos et al. (24)	Outpatient	AN, BN, EDNOS	Adult	Rapid responders had lower scores on the EDE-Q at post-treatment, were more likely to achieve full remission, and required fewer treatment sessions than non-rapid responders
Thompson-Brenner et al. (25)	Outpatient	BN	Adult	Both percentage change in purging frequency and percentage change in depressive symptoms at week 4 were associated with remission at termination, but only change in depressive symptoms at week 4 predicted remission at 6-month follow-up
Wonderlich et al. (26)	Outpatient	BN	Adult	Both CBT-E and integrative cognitive-affective therapy were associated with improvements in bulimic symptoms and other outcome measures and no differences between the two treatments were observed at the end of treatment or at 4-month follow-up
Zipfel et al. (27)	Outpatient	AN	Adult	Increases in body mass were observed in patients treated with CBT-E, focal psychodynamic therapy, or optimized treatment as usual with no differences between groups at the end of treatment or at 12-month follow-up; no group differences were observed on the Eating Disorder Inventory-2; at the end of treatment, patients assigned to CBT-E had lower symptom severity scores based on an expert interview as compared to optimized treatment as usual; at 12-month follow-up, patients assigned to focal psychodynamic therapy had a higher recovery rate as compared with optimized treatment as usual

*AN, anorexia nervosa; BN, bulimia nervosa; BED, binge eating disorder; EDNOS, eating disorder not otherwise specified; EDE-Q, eating disorder examination-questionnaire*.

However, limitations of this study have to be addressed as well, such as lack of a control group, which would allow evaluating the genuine impact of CBT-E on weight gain and eating disorder pathology. Moreover, an adapted form of CBT-E was used, which included many elements that do not typically form the standard treatment approach. In particular, the structure of an inpatient treatment with its multimodal treatment offers, its long duration (20 weeks), and involvement of parents in the treatment may also contribute to positive long-term treatment outcomes, independent of CBT-E-specific effects. Moreover, during the follow-up period, most patients received outpatient treatment, which probably influenced long-term outcome.

Therefore, future research urgently needs to include a control group and control for additional treatment effects after discharge from the inpatient unit. Moreover, randomization of patients to different treatments seems necessary in order to prevent a selection of highly motivated patients, which may bias outcome interpretation. Notwithstanding these limitations, this study adds to the growing evidence of effectiveness of CBT-E in the treatment of AN and may constitute a basis for and will inspire future research by extending CBT-E to the treatment of adolescent AN patients, which hopefully will improve long-term treatment success in this population.

## Conclusion and Future Directions

In recent years, research efforts aiming at optimizing treatment outcome of adolescent AN patients have intensified and proved as fruitful and promising. Besides CBT-E for adolescents in different settings described above, other therapies have been adapted from adult AN patients to juvenile settings as well, for example, cognitive remediation therapy (CRT) (29, 30). Using CRT, improvements in cognitive flexibility could be shown and, in adults, those patients who particularly exhibited low cognitive flexibility prior to treatment showed the largest decreases in eating disorder pathology (31).

Other treatment strategies focus on parents. For example, in an ongoing study, parent-focused treatment is compared with family based treatment strategies (32). Recent research has also compared the effectiveness of different treatment settings: day-patient treatment after short inpatient care proved to be as effective as the more costly continued inpatient treatment (33). Another approach in this line of research is the investigation of home treatment, which is supposed to help overcoming the difficulties that usually arise with the transition from inpatient treatment discharge to the return home (34).

Considering that with the standard treatment, two-thirds of patients can be reached and a considerable number of additional treatment strategies exist, future research should also address the question which patients may profit from which additional treatment form. To date, there are only few studies, in which predictors of treatment success as a function of treatment type were investigated. In a recent review, for example, only one study was identified, in which different treatment types were compared in this regard in adolescents with AN (35). It was found that individuals with higher levels of eating-related obsessionality and eating disorder psychopathology benefited more from family based therapy than individual therapy (36). Other studies on predictors of treatment success, however, focused on adults and/or other eating disorders, treatment types, and outcomes (35). Hence, future research efforts need to generate consistent definitions of treatment outcome, apply multivariate predictor models and differentiate between specific eating disorder diagnoses and treatment types, such as family based interventions versus CBT-E. Identifying characteristics of patients who may benefit more from one treatment form or the other would help to better individualize treatment strategies and may prevent hospitalization.

## Conflict of Interest Statement

The authors declare that the research was conducted in the absence of any commercial or financial relationships that could be construed as a potential conflict of interest.
